# Occurrence and risk assessment of trace metals and metalloids in sediments and benthic invertebrates from Dianshan Lake, China

**DOI:** 10.1007/s11356-017-9069-3

**Published:** 2017-05-05

**Authors:** Yan Wu, Yihui Zhou, Yanling Qiu, Da Chen, Zhiliang Zhu, Jianfu Zhao, Ǻke Bergman

**Affiliations:** 1 Key Laboratory of Yangtze River Water Environment (Ministry of Education), College of Environmental Science and Engineering, Tongji University, Shanghai, 200092 China; 2 Department of Environmental Science and Analytical Chemistry, Stockholm University, -10691 Stockholm, SE Sweden; 3 State Key Laboratory of Pollution Control and Resource Reuse, College of Environmental Science and Engineering, Tongji University, Shanghai, 200092 China; 4 School of Environment, Guangzhou Key Laboratory of Environmental Exposure and Health, and Guangdong Key Laboratory of Environmental Pollution and Health, Jinan University, Guangzhou, 510632 China; 5 Swedish Toxicology Sciences Research Center (Swetox), Forskargatan 20, -15257 Södertälje, SE Sweden

**Keywords:** Dianshan Lake, Yangtze River Delta, Trace metal and metalloid, Bioaccumulation, Risk assessment

## Abstract

The present study measured concentrations of Cr, Ni, Cu, Zn, As, Cd, Sb, and Pb in surface sediments and two benthic invertebrate species (*Anodonta woodiana* and *Bellamya aeruginosa*) collected from Dianshan Lake, located in the Yangtze River Delta. The Dianshan Lake acts as one of the most important drinking water sources to Shanghai, the biggest city in China. Concentrations of trace metals and metalloids ranged from 0.04 mg/kg for Cd to 288.0 mg/kg for Zn. Substantial bioaccumulation in invertebrates was observed for Zn and Cu based on the biota-sediment accumulation factor (BSAF) measurements. The results revealed that concentrations of metals and metalloids in sediments from Dianshan Lake were at the lower end of the range of levels found in other regions of China. The assessment of three significantly inter-related evaluation indices, including the geo-accumulation Index (I_geo_), potential ecological risk factor (Er^i^), and mean probable effect concentration quotients (Q_m-PEC_), suggested that sediment-associated trace elements exhibited no considerable ecological risks in the studied watershed. However, the target hazard quotient and hazard index analysis suggested that selected elements (particularly As) accumulation in edible tissues of benthic invertebrates could pose potential health risks to local populations, especially fishermen. Given that wild aquatic organisms (e.g., fish and bivalves) constitute the diet of local populations as popular food/protein choices, further investigations are needed to better elucidate human health risks from metal and metalloid exposure via edible freshwater organisms.

**Electronic supplementary material:**

The online version of this article (doi:10.1007/s11356-017-9069-3) contains supplementary material, which is available to authorized users.

## Introduction

As a result of rapid urbanization and industrial development, elevated concentrations of trace metals and metalloids in freshwater systems have drawn mounting public concerns and been considered as a major threat to the world’s water resources (Hou et al. 2013; Duzgoren-Aydin 2007; Mwanamoki et al. 2015). Various biogeochemical processes through water-sediment exchange could result in remobilizations of trace elements into surrounding environment, and consequently elevate their concentrations to a toxic level for aquatic biota (Vukosav et al. 2014; Schaller et al. 2011), and generally metals or metalloids in their reducible forms are more likely to pose potential risk to ecosystem (Babula et al. 2008). Sediments usually act as both sinks and potential secondary sources of contaminants to water columns or organisms in aquatic environments (Guo et al. 2006; Yang et al. 2014a). Measurement of trace element concentrations in sediments efficiently reflects the extent of contemporary or long-term anthropogenic effects on aquatic ecosystems (Fu et al. 2014). Mussels have been considered a good sentinel organism to indicate the pollution status in freshwater or estuarine ecosystem due to their sedentary nature, abundance globally, and potency to accumulate contaminants via filtrations of ambient water as well as suspended particulate matter (Birch and Apostolatos 2013; Besada et al. 2011; Tsangaris et al. 2013; Baudrimont et al. 2005). Contaminant burdens in benthic invertebrates (e.g., mussels and snails) reflect time-integrated exposure scenarios in water and benthic environments, thus ideal for monitoring anthropogenic pollution in aquatic systems (Kong et al. 2016). These organisms have also been extensively used to interpret potential impact of contamination on other aquatic biota, given their fundamental trophic status in the food webs (Bian et al. 2008; Joksimovic et al. 2011; Ma et al. 2010). Additionally, bivalves are popular food sources in a number of regions worldwide, and hence their edible soft tissues may represent a potential exposure route of contaminants to humans (Storelli 2008; Usero et al. 2004; Llobet et al. 2003).

Previous studies suggested that the ecological risks from heavy metals (Cr, Cu, Ni, Pb, Zn, and Cd) contamination in soil from the Yangtze River Delta (YRD) and sediments from Taihu Lake were moderate (Hang et al. 2009; Zhou et al. 2016; Fu et al. 2013). However, sediments collected from the Yangtze River near Nanjing city and Changshu city were severely polluted by heavy metals (Fu et al. 2013). A report on metal contamination in sediments from Shanghai indicated that the concentrations of cadmium (Cd), copper (Cu), lead (Pb), and zinc (Zn) were elevated and strongly associated with the density of road network (Yang et al. 2014b). Arsenic (As, mean: 13.2 μg/L), and Cd (mean: 4.7 μg/L) in surface water from the Nanjing section of the Yangtze River exhibited elevated carcinogenic risk and may cause adverse effect on local residents (Wu et al. 2009). Additionally, potentially harmful health effects on the YRD residents via consumption of freshwater fish, benthic organisms, and rice should not be overlooked according to previous studies (Hang et al. 2009; Fu et al. 2013; Chi et al. 2007; Tao et al. 2012; Kong et al. 2016).

The important aquatic systems in the YRD region, such as the Tai Lake and Chao Lake, have been extensively studied for trace metal contamination in sediments and organisms at various trophic levels (Li et al. 2013; Fu et al. 2013; Zeng et al. 2012; Tao et al. 2012; Chi et al. 2007; Kong et al. 2016). However, the Dianshan Lake received little investigations, and therefore we selected eight commonly studied metals and metalloids to elucidate the contamination levels of trace elements in the benthic environment of Dianshan Lake The present study aimed to: (1) measure concentrations of eight trace elements including five obligatory toxic elements (As, Cd, Pb, Sb, and Ni) and three essential elements (Cu, Zn, and Cr) in sediments, mussels (*Anodonta woodiana*), and pond snails (*Bellamya aeruginosa*); (2) analyze geographical distributions and ecological risks of the trace metals and metalloids in the sediments; and (3) evaluate bioaccumulation of the selected elements in benthic organisms and the associated impact on human health.

## Materials and methods

### Study site description

Dianshan Lake is located at the boundary between Shanghai and Jiangsu Province in the Yangtze River Delta (YRD) of China. It is one of the most important drinking water and aquatic product (e.g., carps, catfish, eels, mussels, and snails) sources to the metropolitan area of Shanghai, the biggest metropolis in China. It is approximately 60 km west from downtown Shanghai and covers an area of 62 km^2^. Along with an increasing economic prosperity in Shanghai, its surrounding aquatic systems have been subjected to substantial impacts by human activities, receiving a large variety of organic chemicals and metals from various sources, such as wastewater discharges, urban runoff, and air deposition (Pan and Wang 2012).

### Sampling

**Fig. 1:**
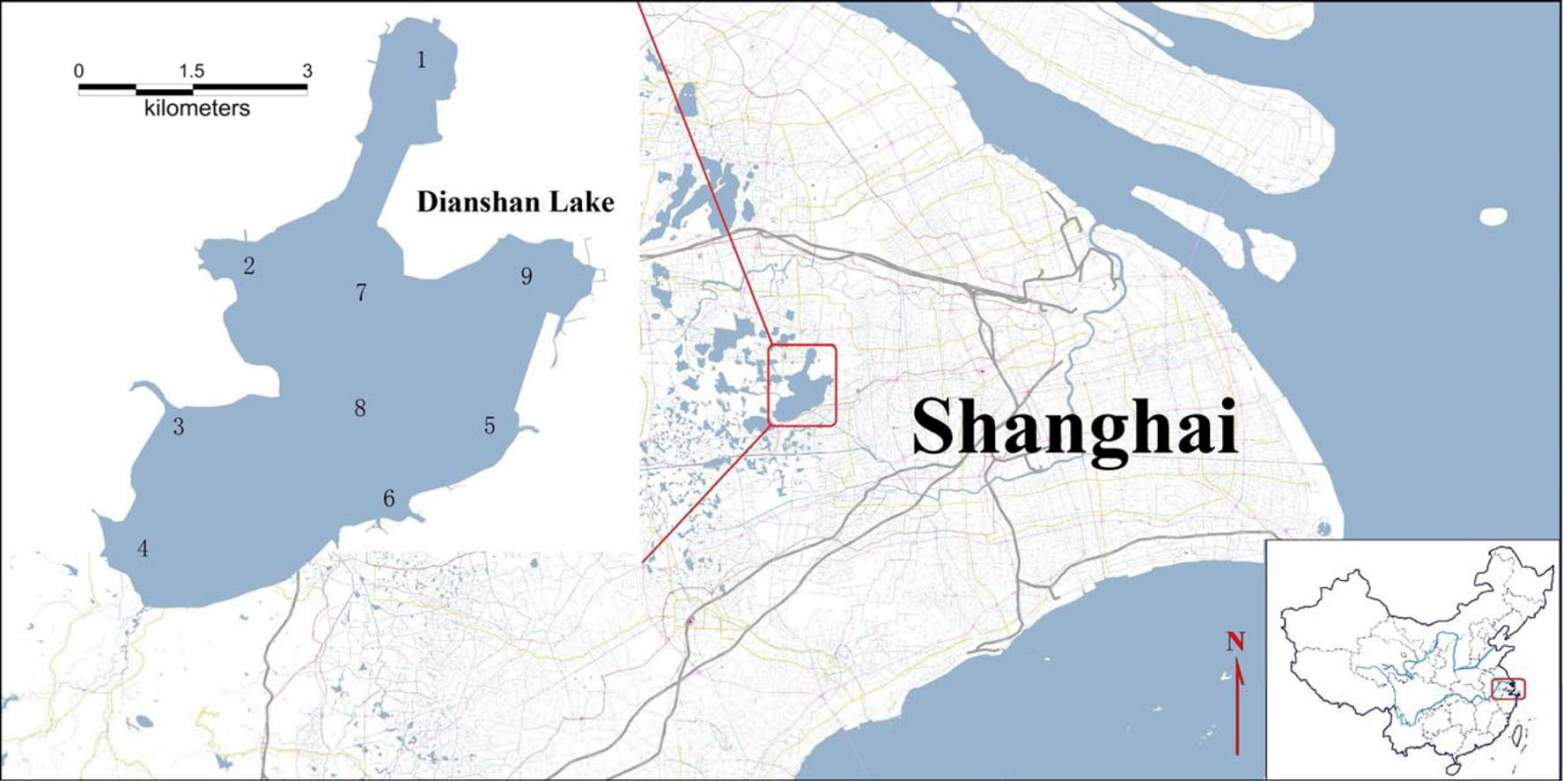
Sampling sites at Dianshan Lake

Sediment samples were collected from nine sites in Dianshan Lake (Fig. 1) in August 2012. Sites 2 Dazhushe (DZS), 3 Jishuigangqiao (JSGQ), and 4 Baishiji (BSJ) were located in the inflow waterways. Site 5 Xizha (XZ) and 6 Dianfeng (DF) were located in the outflow waterways. Site 9 Hudongbei (HDB) was at a water park where a number of recreation facilities made of stainless steel were located. Site 1 Qiandungang (QDG) was located in northern part of the Dianshan Lake and functioned as an aquaculture area. Sites 7 Zhaotianhu (ZTH) and 8 Sihaohangbiao (SHHB) were located in the middle of the lake. Surface sediments, approximately 10 cm in depth from the surface, were sampled with a core sampler (XDB0209, Jiepu Aobo (Beijing) environmental protection engineering equipment Co., Ltd.) and kept in sampling bags. Four sub-samples, approximately 50 cm away from each other, were sampled at each site and mixed to make one homogenate. In addition, 2–5 individual mussels (*Anodonta woodiana*) and 30–120 individual pond snails (*Bellamya aeruginosa*) were collected at each sediment sampling site. The body sizes (with shell) of mussels and pond snails were 110–230 and 9–24 mm, respectively. These two benthic species are abundantly present in the Dianshan Lake as fundamental components of aquatic food webs, as well as popular food items consumed by residents in the YRD region. All samples were stored at −80 °C prior to analysis.

### Sample treatment and instrumental analysis

The collected mussels and snails were subject to depuration in Milli-Q water for 24 h, and then their soft tissues were removed from the shells using pre-cleaned Teflon scoop. Two shell-free mussels and 20 snails from each site were pooled to make a composite per species. Sediment and tissue composites were homogenized and freeze-dried for 48 h. The freeze-dried sediment samples were milled, sieved through a nylon mesh with a diameter of 0.150 mm. Total organic carbon (TOC) of sieved sediments was determined using a TOC analyzer (Shimadzu TOC-VCPN). The analytical procedure of trace metals and metalloids in sediments and tissues was built based on the methodologies previously reported with slight modifications (Birch and Apostolatos 2013; Mwanamoki et al. 2015). Dried tissue composites were ground into powders. Approximately 1 g of pretreated sediment or 0.3 g of tissue was digested by 15 mL of a mixture of hydrochloric acid (HCl), nitric acid (HNO_3_), and hydrofluoric acid (HF) (1:3:1, v:v:v) using a microwave digester (Shanghai Xintuo Analytical Instruments Co., LTD, China). At a constant microwave power of 2400 kW, the oven was programmed as follows: starting at ambient temperature for 1 min, ramped at 65 °C/min to 150 °C, and held for 150 s; ramped at 15 °C/min to 180 °C and held for 150 s; ramped at 10 °C/min to 200 °C and held for 150 s; and finally ramped at 10 °C/min to 220 °C, held for 15 min (31.5 min in total). After digestion, the solution in vessel was concentrated at 110 °C till approximately 1–2 mL, and then spiked with 3 mL of concentrated nitric acid. The resulting mixture was evaporated at 110 °C again to completely remove remaining hydrofluoric acid. The concentrated solution was filtrated through quantitative filter paper, transferred into a 25 mL volumetric flask, and then diluted with 4% nitric acid solution. The treated samples were then preserved in clean polypropylene tubes prior to instrumental analysis. Further dilution might be needed if element concentrations were beyond the range of the concentrations of calibration standards.

Concentrations of As, Cd, Cr, Cu, Ni, Pb, Sb and Zn in samples, blanks, certified reference material (CRM), and external standards were measured by inductively coupled plasma interfaced with mass spectrometry (Agilent ICP-MS7700, Agilent Technologies, USA). Injections were performed in the splitless mode. The speed of peristaltic pump and temperature in the pre-mixer were set to 0.1 rps (round per second) and 2 °C, respectively. High-purity argon functioned as both carrier and make-up gas at a constant flow of 0.7–0.75 and 0.2–0.3 L/min, respectively. High-purity helium acted as reaction gas at 0.08 L/min. A Full Quant analyzing model was applied to the detection of all elements studied. The integral time was 3 s for As, Cd and Sb, and 1 s for Pb, Cu, Zn, Ni, and Cr. Quantification was carried out via external standard method.

### QA/QC

Accuracy of the analytical method was validated using the stream sediment CRM (GBW07303a) purchased from the National Research Center for CRMs (Beijing, China). The recoveries of selected metals and metalloids in the sediment CRM were acceptable with all values greater than 75% (Table S1). The recovery of chromium could be improved by digesting with sulfuric acid (H_2_SO_4_) or phosphoric acid (HClO_4_), However, neither of them was added due to safety concerns during microwave digestion.

A blank and a CRM sample were analyzed along with every batch (*n* = 7) of sediments or tissues. Each authentic sample was treated in triplicates to check for the analytical precision, and the relative standard deviations of triplicate samples were less than 20%. The limit of quantifications (LOQs, ng/mL), defined as a concentration three times the limit of detection (LOD) computed by the ICP-MS, were 8.87, 1.33, 3.56, 22.71, 0.16, 0.026, 0.0015, and 1.65 for Cr, Ni, Cu, Zn, As, Cd, Sb, and Pb, respectively.

### Risk assessment approaches

Contamination levels and potential ecological risks of trace elements in sediments were assessed using the geo-accumulation Index (I_geo_), potential ecological risk factor (Er^i^), and mean probable effect concentration quotients (Q_m-PEC_) (Muller 1969; Hakanson 1980; MacDonald et al. 2000). Potential human health risks via edible tissues of mussels and pond snails were assessed using the target hazard quotient (THQ) and hazard index (HI) (USEPA 1989). These approaches are introduced in detail in Supplementary data.

In order to estimate metal and metalloid accumulation potential in benthic organisms, biota-sediment accumulation factors (BSAFs) were determined according to the following formula (Szefer et al. 1999):

1 $$ \mathrm{BSAF}={C}_b/{C}_s $$

where *C*_*b*_ and *C*_*s*_ are the mean concentrations in benthic organisms and sediments, respectively.

All data were statistically analyzed using OriginPro 9.0 (OriginLab Corporation). A significance level *α* = 0.05 was applied. The calculations and evaluation criteria for I_geo_, Er^i^, Q_m-PEC_, THQ and HI were described in Supplementary Data.

## Results and discussion

### Trace metal and metalloid concentrations in sediments

Concentrations of trace elements in sediments from Dianshan Lake are summarized in Table 1, with ranges of 41.4–75.3, 23.3–54.3, 16.7–94.7, 51.7–304.4, 5.8–17.2, 0.03–0.11, 0.65–2.7, 15.8–28.3 μg/kg dw for Cr, Ni, Cu, Zn, As, Cd, Sb, and Pb, respectively. In general, site 1 appeared to be the most contaminated site with the highest concentrations of Ni, Cu, Zn, Cd, Sb, and Pb found across the Lake, while site 5 contained the lowest concentrations of Cr, Ni, Cu, As, Sb, and Pb in the sediment. The highest concentrations of Cr and As were found at sites 9 and 6, respectively. Compared to global data on freshwater sediments, concentrations of Cd and Pb in Dianshan Lake sediments were lower than 25th percentile of the gathered sediment data summarized by Fu et al. (2014) and Ma et al. (2013). Concentrations of Cr, Cu, As, and Zn were lower than the medium level (25–50%), but Ni concentration was higher than the median. He et al. (2012) has reviewed Sb pollution in China and reported that the Sb concentration in sediments throughout the Yangtze River watershed ranged from 0.50 to 2.7 mg/kg dw. Sediments in Dianshan Lake contained a geometric mean Sb concentration of 1.12 mg/kg, lower than the majority of lakes in Southern China, but higher than the Songhua River in Northern China which had Sb concentrations between 0.18 and 0.57 mg/kg. Studies of Sb contamination outside China usually focused on extreme cases where high Sb concentrations were found in sediments near antimony mines. Thus, the data were not comparable to our findings.

**Table 1:** Trace metal concentrations ( ± standard deviation; mg/kg dry weight) in sediments and benthic invertebrates from Dianshan Lake

Sediments									
Location	Site	Cr	Ni	Cu	Zn	As	Cd	Sb	Pb
QDG	1	64.79 ± 2.54	51.00 ± 2.91	88.96 ± 4.98	288.0 ± 15.6	13.26 ± 0.48	0.105 ± 0.005	2.30 ± 0.37	27.72 ± 0.74
DZS	2	64.02 ± 4.60	38.41 ± 2.51	24.72 ± 1.89	75.04 ± 5.45	10.12 ± 0.40	0.056 ± 0.012	0.95 ± 0.05	20.53 ± 1.12
JSGQ	3	55.95 ± 5.65	34.32 ± 3.40	28.18 ± 2.72	72.22 ± 6.64	12.01 ± 1.08	0.055 ± 0.004	1.19 ± 0.10	20.64 ± 1.45
BSJ	4	57.51 ± 3.14	33.11 ± 2.19	22.85 ± 1.35	55.97 ± 2.87	10.48 ± 0.05	0.040 ± 0.012	1.31 ± 0.42	19.98 ± 0.35
XZ	5	48.61 ± 7.46	27.99 ± 4.56	19.61 ± 2.87	61.98 ± 10.3	6.561 ± 0.76	0.053 ± 0.010	0.83 ± 0.24	17.14 ± 1.37
DF	6	60.86 ± 7.17	38.57 ± 4.29	24.42 ± 2.59	59.67 ± 7.04	15.58 ± 1.58	0.049 ± 0.002	1.00 ± 0.10	21.45 ± 1.64
ZTH	7	61.42 ± 4.55	38.18 ± 2.87	40.89 ± 2.98	77.01 ± 4.84	10.72 ± 0.81	0.067 ± 0.002	1.30 ± 0.07	23.84 ± 1.06
SHHB	8	61.67 ± 5.31	35.61 ± 3.31	23.96 ± 2.34	74.87 ± 7.02	6.973 ± 0.55	0.053 ± 0.003	0.90 ± 0.14	21.11 ± 0.52
HDB	9	70.68 ± 4.03	44.27 ± 2.64	32.70 ± 2.04	94.82 ± 7.87	12.31 ± 0.41	0.055 ± 0.006	0.86 ± 0.04	24.96 ± 0.97
Benthic invertebrates									
Species		Cr	Ni	Cu	Zn	As	Cd	Sb	Pb
Pooled mussels		18.49 ± 2.18	10.27 ± 0.63	16.72 ± 1.33	684.9 ± 52.9	3.02 ± 0.17	0.148 ± 0.055	0.085 ± 0.007	1.18 ± 0.15
Pooled pond snails		25.70 ± 1.33	14.47 ± 0.73	90.03 ± 7.76	157.8 ± 17.3	1.95 ± 0.22	0.135 ± 0.023	0.090 ± 0.012	2.40 ± 0.30

The principal component analysis (PCA) was further employed to determine the data structure and facilitate data interpretation. Two factors were extracted and accounted for 89.4% of the total variance in the dataset (Fig. S1). According to the biplot, some sites (i.e., sites 1, 5, and 9) differed from the others in the contamination of individual element. The relatively higher concentration of Cr at site 9 (located in a water park) compared to other sites may be due to a number of recreational facilities located near the site. These facilities were generally made of stainless steel (chromium as main alloy element). Elevated concentrations of Sb, Cu, Zn, and Cd at site 1 were probably because it was located in a semi-closed aquaculture area, where Zn has been used as an additive to fish food and copper sulfate (CuSO_4_) as a disinfector to control disease and cyanobacteria. Low metal and metalloid concentrations at site 5 may be due to dilution effect as the site was located in the outflow waterway of the lake. By contrast, site 6 was located at another outflow point, but its sediments contained high concentrations of As (peak value), Ni, and Pb. Given that an expressway is adjacent to site 6, vehicle exhausts may contribute metals to this particular site. Previous studies suggested that fossil fuel consumption in automobile engine was one of the major anthropogenic sources of As, Pb, and Ni in urban areas (Talebi and Abedi 2005; Thomaidis et al. 2003; Lagerwerff and Specht 1970).

**Fig. 2:**
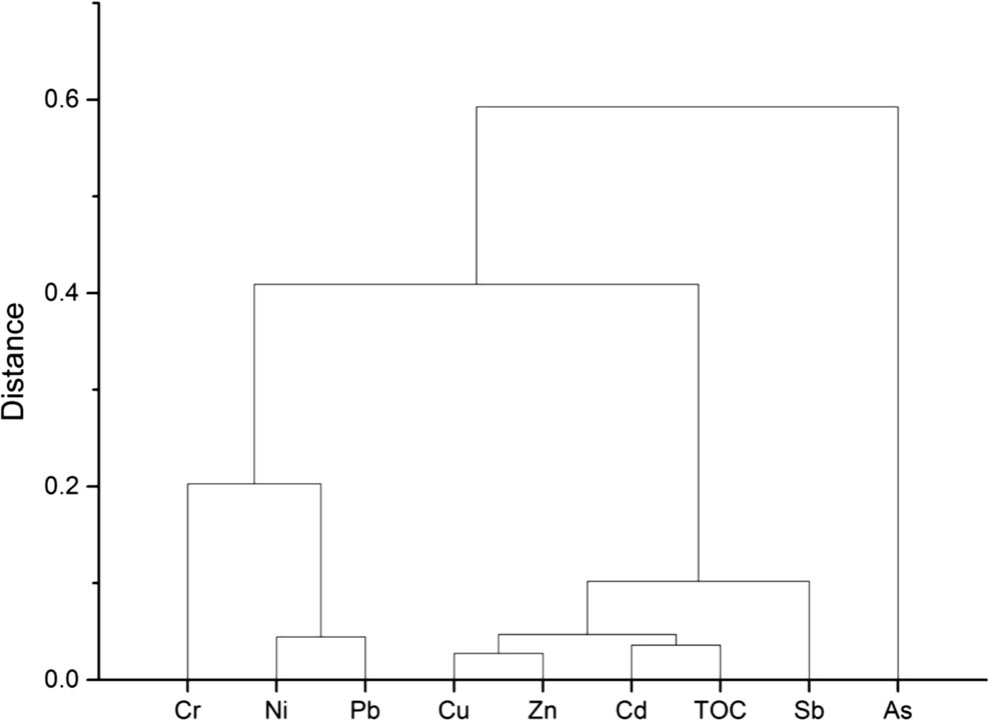
Dendrogram of hierarchical cluster analysis for trace metals in sediments

According to the dendrogram of Hierarchical Cluster Analysis (Fig.  2), Cu, Zn, and Cd are closely associated with each other and form a cluster along with Sb. Ni and Pb form another cluster together with Cr, while a great distance exists between As and other elements. Variables contributing alike information are grouped together, indicating that they may be correlated and may potentially originate from similar pollution sources (Fu et al. 2014; Liu et al. 2016; Nam et al. 2008; Liu et al. 2003). Zhang et al. (2009) reported that Pb and Ni were closely associated with each other in sediments from the Yangtze River intertidal zone. Sources of Cd, Zn, Cu, and Pb in sediments from a catchment of Tai Lake were similar and were demonstrated to be mainly from anthropogenic origins (Bing et al. 2011). Additionally, a close relationship between Cd and TOC observed from the present work is consistent with the findings by Lin et al. (2002) which investigated sediments from the East China Sea continental shelf. A short distance between Cd-TOC and Cu-Zn groups indicates that organic content in sediment may influence concentrations of Cd, Cu, and Zn in our studied waterbody. A weak correlation between As and other elements as shown in the present study was also observed in sediments from the middle and lower reaches of Yangtze River basin (Yi et al. 2011).

### Concentrations and BSAFs of trace metals and metalloids in benthic invertebrates

Concentrations (mean ± standard deviation, mg/kg dw) in mussels collected from Dianshan Lake were 18.5 ± 2.2 (Cr), 10.3 ± 0.6 (Ni), 16.7 ± 1.3 (Cu), 684.9 ± 52.9 (Zn), 3.0 ± 0.2 (As), 0.15 ± 0.06 (Cd), 0.085 ± 0.007 (Sb), and 1.2 ± 0.2 (Pb), while in the pond snails were 25.7 ± 1.3 (Cr), 14.5 ± 0.7 (Ni), 90.0 ± 7.8 (Cu), 157.8 ± 17.3 (Zn), 1.9 ± 0.2 (As), 0.14 ± 0.02 (Cd), 0.09 ± 0.01 (Sb), and 2.4 ± 0.3 (Pb) (Table 1). Contaminants with a BSAF greater than one are prone to migrate from abiotic environment to organisms and might biomagnify along food chains (Szefer et al. 1999; Liu et al. 2010). The BSAF of Cd (2.60) and Zn (8.30) in mussels, as well as that of Cd (2.37), Zn (1.91), and Cu (2.97) in pond snails, indicated the potential of these element species to bioaccumulate in benthic organisms, whereas other elements generally possessed a BSAF value less than one. Unlike Cr, Cd, Ni, Pb, and As which were generally classified as toxic elements, Cu and Zn were essential elements for functions in a number of bivalves including synthesis of hemocyanin and maintenance of catalytic activities, and hence were subject to in vivo regulations by many benthonic organisms (Wang et al. 2013; Rzymski et al. 2014). The High levels of these two elements may be attributed to either high metabolic demand on essential elements or elevated Cu and Zn concentrations in the ambient environment (Rzymski et al. 2014). However, when their concentrations in bivalves reached toxic levels, negative effects from excessive accumulations of Cu and Zn could not be neglected. When compared the BSAF values in the present study to the others, large variations in BSAF value were observed among elements or organisms, suggesting species-, or system-specific bioaccumulation (Table 2). For example, the BSAF of Cu, Zn, and Cd reported in various studies ranged from 0.14 to 75, 0.43–57, and 0.41–136, respectively (Table 2). Nevertheless, some general patterns can be inferred from the comparison. Cd and Cu are generally bioavailable to benthic organisms regardless of species and locations, whereas Cr, Ni, and Pb tend to absorb on sediments and have limited bioavailability in organisms.

**Table 2:** Comparison of concentrations (mg/kg dry weight) and BSAF (in parentheses) of trace metals in benthic organisms from Dianshan Lake and other regions

Location	Species	Metal concentration							Reference
		Cr	Ni	Cu	Zn	As	Cd	Pb	
Dianshan Lake, China	*Anodonta woodiana*	18.5 (0.31)	10.3 (0.27)	16.7 (0.55)	685 (8.30)	3.02 (0.29)	0.148 (2.60)	1.18 (0.05)	This study
	*Bellamya aeruginosa*	25.7 (0.43)	14.5 (0.39)	90.0 (2.97)	158 (1.91)	1.95 (0.19)	0.135 (2.37)	2.40 (0.11)	
Tai Lake, China	*Anodonta woodiana*	n.d.	n.d.	11.2 (0.35)	876 (7.60)	13.4 (1.38)	11.9 (36.6)	0.73 (0.03)	Liu et al. (2010)
Maltański Reservoir, Poland	*Anodonta anatine*	0.33 (0.04)	0.06 (0.02)	9.3 (0.82)	42.2 (0.59)	n.d.	0.04 (0.41)	0.15 (0.06)	Rzymski et al. (2014)
	*Anodonta cygnea*	0.64 (0.08)	0.86 (0.20)	5.3 (0.46)	31.1 (0.43)	n.d.	0.08 (0.81)	0.16 (0.06)	
	*Unio tumidus*	1.1 (0.14)	0.77 (0.18)	4.6 (0.40)	51.3 (0.71)	n.d.	0.04 (0.44)	0.21 (0.08)	
North-western Mediterranean, Europe	*Mytilus galloprovincialis*	1.30 (0.0055)	2.00 (2.3)	n.d.	n.d.	n.d.	1.57 (34)	1.22 (0.054)	Lafabrie et al. (2007)
Galician Rias, Atlantic coast, Europe		11.9 (0.17)	4.9 (0.18)	6.8 (0.14)	131 (0.83)	11.6 (0.51)	n.d.	5.7 (0.047)	Beiras et al. (2003)
Lake Balaton, Europe	*Unio pictorum*	1.85 (0.11)	3.16 (0.41)	6.13 (0.43)	164 (6.2)	n.d.	1.44 (6.0)	1.95 (0.035)	Nguyen et al. (2005)
Baltic Sea, Europe	*Mya arenaria*	0.82 (0.027)	n.d.	13.4 (0.67)	269 (3.0)	n.d.	2.35 (2.1)	5.22 (0.12)	Pempkowiak et al. (1999)
	*Astarte borealis*	1.56 (0.052)	n.d.	12.1 (0.60)	185 (2.1)	n.d.	6.14 (5.6)	4.90 (0.11)	
Sg. Sarawak Kanan, Malaysia	*Brotia costula*	n.d.	n.d.	58.3 (52.0)	185 (57.0)	13.7 (12.0)	n.d.	0^a^	Lau et al. (1998)
	*Melanoides tuberculata*	n.d.	n.d.	85.0 (75.0)	110 (34.0)	28.3 (24.0)	n.d.	0^a^	
Tai Lake, China	*Bellamya sp.*	8.62 (0.11)	4.53 (0.076)	95.4 (1.9)	117 (0.87)	n.d.	0.630 (0.57)	2.74 (0.064)	Tao et al. (2012)
Turkish Coast, Black Sea, Europe	*Rapana venosa*	0.730 (0.012)	0^a^	36.2 (1.3)	256 (2.8)	n.d.	15.6 (21)	0^a^	Topcuoğlu et al. (2002)
Mazatlan Bay, Mexico	*Crassostrea iridescens*	0.990 (0.054)	5.41 (0.40)	86.9 (2.5)	1160 (7.2)	n.d.	2.30 (2.6)	2.30 (0.040)	Soto-Jiménez (2001)
Gulf of Oman, Asia	*Pinctada radiata*	2.36 (0.13)	7.02 (3.5)	17.3 (8.7)	n.d.	30.6 (24)	2.73 (136.5)	2.29 (2.9)	de Mora et al. (2004)
Amurskiy and Ussuriyskiy Bays, Sea of Japan	*Crassostrea gigas*	n.d.	2.50 (0.0069)	3950 (1.1)	5280 (0.92)	n.d.	17.6 (3.6)	2.5 (0.00078)	Shulkin et al. (2003)

*n.d.* not detected

^a^Quantifiable in sediments but not in organisms

### Risk assessment of trace metals and metalloids in sediments

**Fig. 3:**
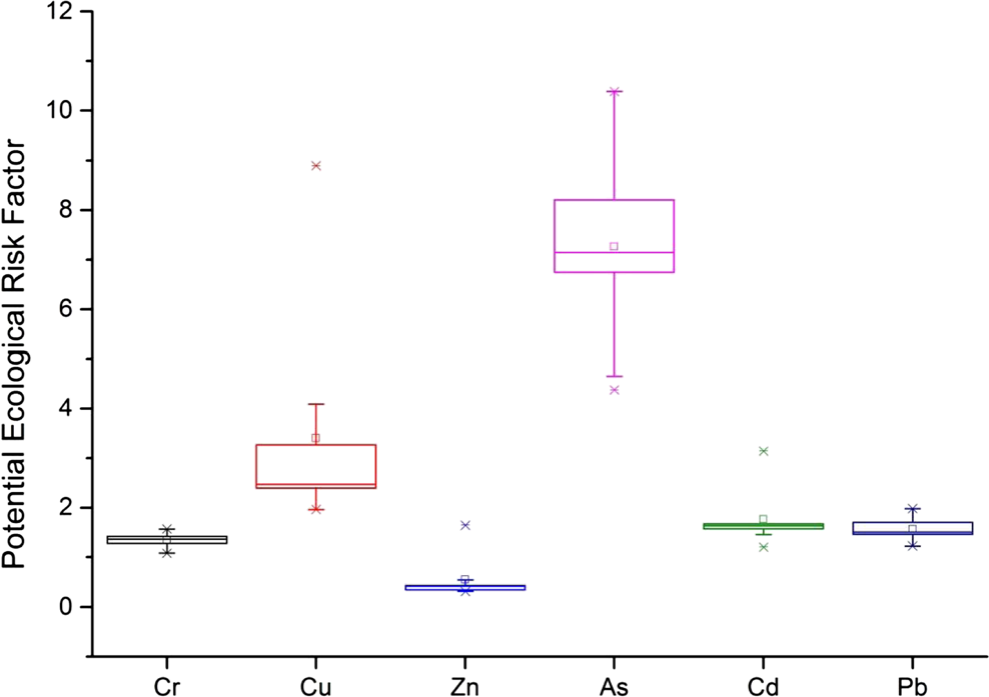
Potential ecological risk factor for trace metals in Dianshan Lake sediments

Three risk assessment indices (I_geo_, E_r_^i^ and Q_m-PEC_) were significantly correlated with each other for each trace metal and metalloid studied (except for Ni and Sb due to lack of preindustrial reference levels), with correlation coefficients greater than 0.98 (*n* = 9, *p* < 0.001). The I_geo_ and E_r_^i^ values for As are greater than those for other elements (Fig. 3 and S2). The I_geo_ values of all elements were generally below zero, except for Cu, Zn, and Sb from site 1 (treated as outliers by box plots). The maximum value of E_r_^i^ is 10.4, corresponding to a high As concentration at site 6. Therefore, according to the interpretation of the geoaccumulation indexes and potential ecological risk factors (Muller 1969; Hakanson 1980), the Dianshan Lake sediments were subject to relatively low trace metal and metalloid contamination, leading to no considerable ecological risk.

The mean PEC quotient of 0.5 is a threshold to classify sediments as either toxic or nontoxic (MacDonald et al. 2000). The Q_m-PEC_ values in the present study are no greater than 0.5 for all sampling sites, indicating the Dianshan Lake sediments were not toxic to benthic organisms (Table 3). This is consistent with the findings based on the I_geo_ and E_r_^i^ assessments. The individual PEC quotient reveals that the risks posed by Ni and Cr in sediments were the greatest, followed by As, Cu, Zn, Pb, and Cd in sequence. Regarding the two metals with the highest individual PEC quotient, bioavailability of Ni and Cr as well as their mobilizations between sediments and overlying water were influenced by several environmental factors, including temperature, benthic oxygen demand, sediment-water flux, and presence of acid-volatile sulfide (Berry et al. 2004; Shine et al. 1998). The mean Q_m-PEC_ of inflow points (sites 2, 3, and 4; mean = 0.296) is similar to those of center points (sites 7 and 8; mean = 0.308) and outflow points (sites 5 and 6; mean = 0.279), but significantly lower than that of the sites intensively affected by human activities (sites 1 and 9; mean = 0.432; *p* = 0.011).

**Table 3:** The probable effect concentration (PEC) quotient for trace metals in Dianshan Lake sediments

Site abbreviation	Site number	Cr	Ni	Cu	Zn	As	Cd	Pb	Q_m-PEC_
QDG	1	0.584	1.049	0.597	0.627	0.402	0.021	0.217	0.500
DZS	2	0.577	0.790	0.166	0.163	0.307	0.011	0.160	0.311
JSGQ	3	0.504	0.706	0.189	0.157	0.364	0.011	0.161	0.299
BSJ	4	0.518	0.681	0.153	0.122	0.318	0.008	0.156	0.279
XZ	5	0.438	0.576	0.132	0.135	0.199	0.011	0.134	0.232
DF	6	0.548	0.794	0.164	0.130	0.472	0.010	0.168	0.326
ZTH	7	0.553	0.786	0.274	0.168	0.325	0.013	0.186	0.329
SHHB	8	0.556	0.733	0.161	0.163	0.211	0.011	0.165	0.286
HDB	9	0.637	0.911	0.219	0.207	0.373	0.011	0.195	0.365

### Assessment of human health risks via edible benthic organisms

**Fig. 4:**
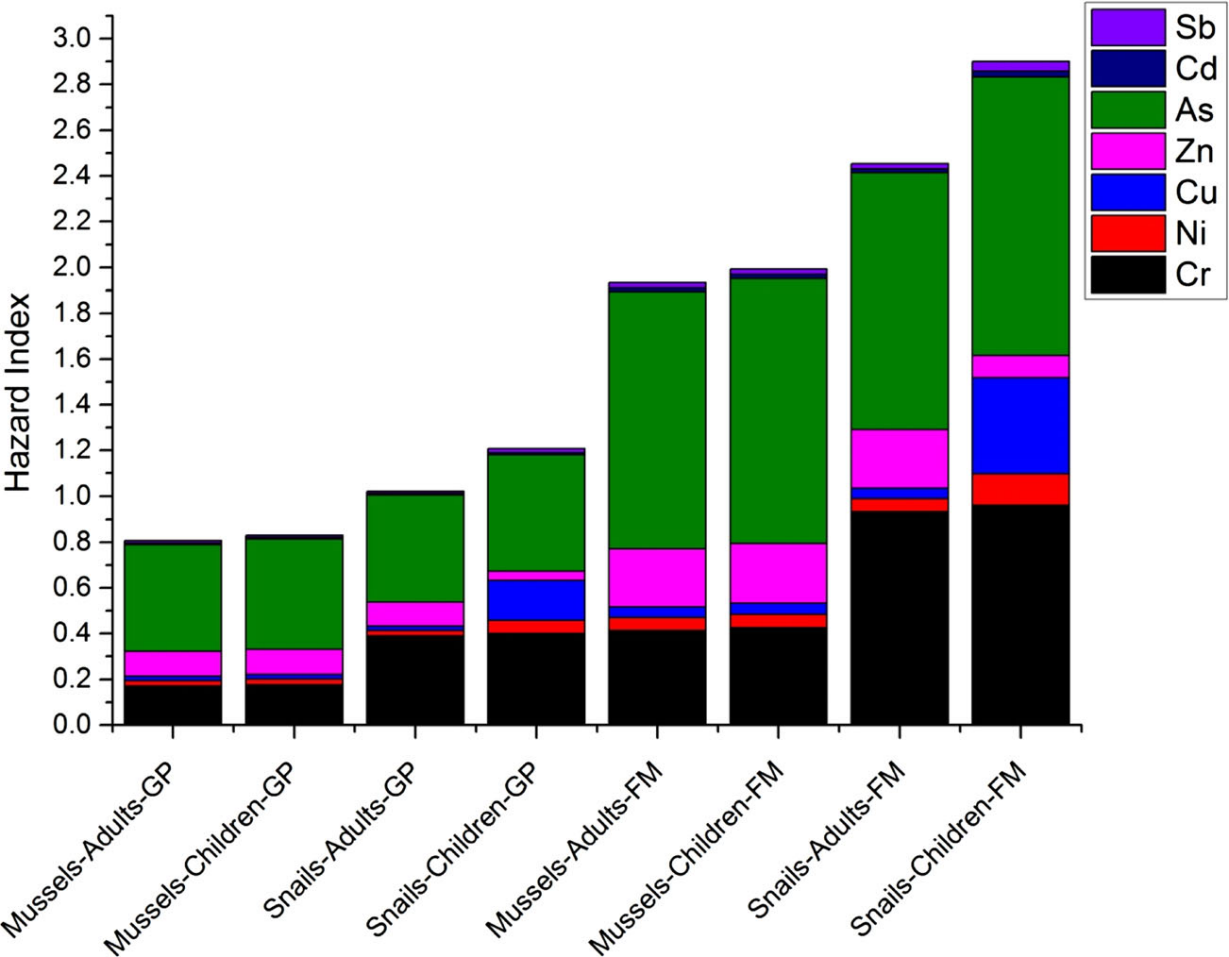
Hazard indexes (HI) of trace metals for general populations (GP) and fishermen (FM)

Two groups of human populations (i.e., general population and fishermen) were assessed for the risk to metal and metalloid exposure via ingestion of benthic organisms. The THQs and HIs for adults and children were calculated for each group (Table S4 and Fig. 4). Due to the difficulty in accounting for pre-existing body burdens of Pb and the lack of threshold levels for health risk evaluations, it is inappropriate to develop reference values for Pb (USEPA 2004). Thus, Pb was not included in the THQ and HI determination. All other elements revealed THQ less than one, except for As which had the THQs of 1.123–1.216 for fishermen. This indicates that fishermen living in the Dianshan Lake basin might be subject to a considerable non-cancer health risk posed by As. It was noteworthy that the bioavailability and physiological toxicity of arsenic depends its chemical forms (Mandal 2002). Arsenic in trivalent state (both inorganic and organic) holds more potency to exert adverse effects on human beings (Paul et al. 2013). Unlike human exposure to As through drinking water which might be subject to rapid elimination via urination, As intake by human via diet could be absorbed by gastrointestinal tract and distributed into body to impair the normal functions (Alamdar et al. 2017).

The hazard indexes for the exposure of general population to mussels were generally less than one, whereas all other HI values exceeded unity (Fig. 4). A HI value greater than 1.0 does not necessarily suggest a likelihood of adverse effects, especially when only one to two substances are responsible for elevating HI values (USEPA 2005, 1989). However, the potential health effects via the consumption of benthic organisms by local people, particularly by fishermen, are worth noticing. Fishermen (both adults and children) living in the Dianshan Lake basin may be subject to a potential health risk through the ingestion of selected benthic organisms. Potential health effects may be more significant for children than adults. Therefore, although the risk assessment indices indicated no considerable ecological risks posed by sediment-associated metals and metalloids, potential adverse health effects on humans (particularly fishermen) via the consumption of contaminated benthic invertebrates should not be neglected. This is mainly due to a relatively high rate of intake of various bivalves as human food in the YRD basin.

## Conclusions

In the present study, the occurrence of eight trace elements in sediments and their bioaccumulation in benthic invertebrates in the Dianshan Lake were investigated. Zn and Cu exhibited substantial bioaccumulation in invertebrates according to the BSAF measurement. The evaluation of different risk assessment indices, including the geo-accumulation index, potential ecological risk factor, and mean probable effect concentration quotients, suggested that sediment-associated metals or metalloids produced no considerable ecological risks in the studied watershed. However, the target hazard quotient and hazard index analysis suggested that metal and metalloid (As in particular) accumulation in bivalves could pose potential health risks to local populations, especially fishermen. Arsenic is the priority pollutant among the selected elements in both sediments and invertebrates, leading to the highest potential ecological risk factor, geoaccumulation index, and target hazard index. Given that wild aquatic organisms (e.g., fish and bivalves) constitute the diet of local populations in the YRD basin as popular food/protein choices, further investigations are needed to better elucidate human health risks via contaminated freshwater organisms in the studied region.

## Electronic supplementary material

ESM 1 (DOCX 226 kb).
