# Supplementary material for: Occurrence and risk assessment of trace metals and metalloids in sediments and benthic invertebrates from Dianshan Lake, China
Source: Environ Sci Pollut Res Int. 2017 May 5;24(17):14847–56. doi: 10.1007/s11356-017-9069-3 (PMC6677693; doi:10.1007/s11356-017-9069-3)
Supplement: Supplementary file 1 — (DOCX 226 kb). [file 11356_2017_9069_MOESM1_ESM.docx]

*Supplementary material*

**Occurrence and Risk Assessment of Trace Metals and Metalloids in Sediments and Benthic Invertebrates from Dianshan Lake, China**

Yan Wu^1,2^, Yihui Zhou^3^, Yanling Qiu*^1^, Da Chen^4^, Zhiliang Zhu^1^, Jianfu Zhao^3^, Ǻke Bergman^2,3,5^

^1^ Key Laboratory of Yangtze River Water Environment (Ministry of Education), College of Environmental Science and Engineering, Tongji University, Shanghai 200092, China

^2^ Department of Environmental Science and Analytical Chemistry, Stockholm University, Stockholm SE-10691, Sweden

^3^ State Key Laboratory of Pollution Control and Resource Reuse, College of Environmental Science and Engineering, Tongji University, Shanghai 200092, China

^4^ School of Environment, Guangzhou Key Laboratory of Environmental Exposure and Health, and Guangdong Key Laboratory of Environmental Pollution and Health, Jinan University, Guangzhou 510632, China

^5^ Swedish Toxicology Sciences Research Center (Swetox), Forskargatan 20, SE-152 57 Södertälje, Sweden

* Corresponding Authors. E-mails: [ylqiu@tongji.edu.cn](mailto:ylqiu@tongji.edu.cn)**Calculations and evaluation criteria for Igeo, Eri, Qm-PEC, THQ and HI**

Geoaccumulation Indexes (I_geo_) pioneered by [Muller (1969)](#_ENREF_6), Potential Ecological Risk Factors (Er^i^) proposed by [Hakanson (1980)](#_ENREF_2) and Mean Probable Effect Concentration Quotients (Q_m-PEC_) suggested by Consensus-Based Sediment Quality Guidelines (SQGs) ([MacDonald et al. 2000](#_ENREF_5)) were calculated to evaluate the trace metal and metalloid contaminations in the sediments from Dianshan Lake. Their calculations are expressed below as:

I_geo_ =log_2_ (C_n_/1.5B_n_) (Eq.1)

where C_n_ is the measured concentration of the element in sediments, and B_n_ is the corresponding geochemical background value. Insertion of constant 1.5 takes into account the natural fluctuations in the content of a chemical in the environment and perceives tiny anthropogenic influences ([Ji et al. 2008](#_ENREF_3)).

E_r_^i^ =T_r_^i^*C_f_^i^=T_r_^i^*C^i^/C_n_^i^ (Eq.2)

where T_r_^i^ is the toxic-response factor for a given substance; C_f_^i^ is the contamination factor; C^i^ is the measured result, and C_n_^i^ is the corresponding preindustrial reference level.

Q_m-PEC_ **=** $\frac{\sum_{1}^{n} {C_{n}}/{C_{consensus-based PEC}}}{n}$ (Eq. 3)

where C_n_ is the measured element concentration, and C_Consensus-Based PEC_ is the corresponding Consensus-Based Probable Effect Concentration. The individual PEC Quotients (PEC-Qs) are summed and divided by the number of PEC-Qs to yield a Q_m-PEC_. There is considerable evidence demonstrating that Q_m-PEC_ could be indicative of presence or absence of toxic impacts with a quantifiable level of confidence ([Long 2006](#_ENREF_4)).

As geochemical background values of Dianshan Lake was no available, average content of metals and metalloids in sedimentary shale of the earth’s crust ([Turekian and Wedepohl 1961](#_ENREF_8)) were adopted. The toxic-response factors, preindustrial reference levels, geochemical background values and Consensus-Based Probable Effect Concentrations applied in present study are listed in Table S2. Terminologies used to describe I_geo_ and E_r_^i^ are given in Table S3. A mean PEC quotient of 0.5 is usually treated as a robust threshold applied into classifications of sediment samples as both toxic and non-toxic ([MacDonald et al. 2000](#_ENREF_5)).

The risks mussels and snails pose to the local consumers were extrapolated using Target Hazard Quotient (THQ) ([Chien et al. 2002](#_ENREF_1)) and Hazard Index (HI) ([USEPA 1989](#_ENREF_9)). Due to the geographical proximity between Dianshan Lakes and Taihu Lake, dietary habits of people around two lakes were assumed to be alike. Therefore, the parameters suggested by [Tao et al. (2012)](#_ENREF_7) were employed. THQ was calculated by:

THQ=$\frac{E_{f}E_{d}F_{ir}C}{R_{fd}W_{ab}T_{a}}*{10}^{-3}$ (Eq. 4)

where E_f_ is exposure frequency (365 day/year); E_d_ is the exposure duration (70 years); F_ir_ is the rate of ingesting *Anodonta woodiana* or *Bellamya aeruginosa* (general population: 16.7 g/day for adults and 9.0g/day for children; fishermen: 40.1 g/day for adults and 21.6 g/day for children); C is the metal or metalloid concentration in zoobenthos (mg/kg, ww); R_fd_ is the non-cancer reference dose (mg/kg/day) published by [USEPA (1997)](#_ENREF_10) and [USEPA (2013)](#_ENREF_11); W_ab_ is the average body weight (63.1 kg for adults; 33 kg for children); T_a_ is the average exposure time for non-carcinogens (365 day/year*70 years). Provided a THQ is larger than one, populations exposed to the zoobenthos will undergo non-cancer health risks, while if it is below one there will be no appreciable health risks.

A hazard index (HI) for a specific receptor/pathway combination is generated adding the THQs together ([Zheng et al. 2007](#_ENREF_12)), as described below:

HI=$\sum THQ$ (Eq. 5)

When the HI exceeds unity, there may be concern on adverse health effects. For multiple chemical exposures, the HI can also pass unity even though no single chemical exposure has been beyond its R_fD_ ([USEPA 1989](#_ENREF_9)).

Table S1

Recoveries of trace metals and metalloids in sediment CRM (n = 6).

|  | Certified concentration  (mg/kg dry weight) | Observed concentration ± SD  (mg/kg dry weight) | Recovery (%) |
| --- | --- | --- | --- |
| Cr | 48 | 36.64 ± 3.19 | 77.08 |
| Ni | 20 | 19.67 ± 1.40 | 97.71 |
| Cu | 202 | 188.5 ± 16.4 | 93.09 |
| Zn | 102 | 96.25 ± 7.59 | 94.16 |
| As | 16.7 | 16.53 ± 1.49 | 98.32 |
| Cd | 0.50 | 0.4791 ± 0.0664 | 95.15 |
| Sb | 3.8 | 3.769 ± 0.290 | 96.88 |
| Pb | 45 | 43.50 ± 3.89 | 95.91 |

Table S2

T_r_^i^, C_n_^i^ (mg/kg dw), B_n_ (mg/kg dw) and Consensus-Based PEC (mg/kg dw).

|  | Cr | Ni | Cu | Zn | As | Cd | Sb | Pb |
| --- | --- | --- | --- | --- | --- | --- | --- | --- |
| Toxic response Factor (T_r_^i^) | 2 | \ | 5 | 1 | 10 | 30 | \ | 5 |
| Preindustrial Reference Level (C_n_^i^) | 90 | \ | 50 | 175 | 15 | 1.0 | \ | 70 |
| Geochemical Background Values (B_n_) | 90 | 68 | 45 | 95 | 13 | 0.3 | 1.5 | 20 |
| Consensus-Based PEC | 111 | 48.6 | 149 | 459 | 33 | 4.98 | \ | 128 |

\: no data available

Table S3

Terminology describing geoaccumulation index (I_geo_) and potential ecological risk factor (E_r_^i^).

| GI | Contamination level | PERF | Potential ecological risk |
| --- | --- | --- | --- |
| Igeo≤0 | Practically uncontaminated | Eri<40 | Low potential ecological risk |
| 0<Igeo<1 | Uncontaminated to moderately contaminated | 40≤Eri<80 | Moderate potential ecological risk |
| 1<Igeo<2 | Moderately contaminated | 80≤Eri<160 | Considerable potential ecological risk |
| 2<Igeo<3 | Moderately to heavily contaminated | 160≤Eri<320 | High potential ecological risk |
| 3<Igeo<4 | Heavily contaminated | 320≤Eri | Very high potential ecological risk |
| 4<Igeo<5 | Heavily to extremely contaminated |  |  |
| 5<Igeo | Extremely contaminated |  |  |

Table S4

The target hazard quotient (THQ) of trace metals and metalloids in zoobenthos from Dianshan Lake

| General Population | | | | | | | |
| --- | --- | --- | --- | --- | --- | --- | --- |
|  | Cr | Ni | Cu | Zn | As | Cd | Sb |
| Mussels-Adults | 0.172 | 0.024 | 0.019 | 0.106 | 0.468 | 0.007 | 0.010 |
| Pond snails-Adults | 0.389 | 0.024 | 0.019 | 0.106 | 0.468 | 0.007 | 0.010 |
| Mussels-Children | 0.177 | 0.025 | 0.020 | 0.109 | 0.482 | 0.007 | 0.010 |
| Pond snails-Children | 0.400 | 0.056 | 0.175 | 0.041 | 0.507 | 0.011 | 0.017 |
| Fishermen | | | | | | | |
|  | Cr | Ni | Cu | Zn | As | Cd | Sb |
| Mussels-Adults | 0.412 | 0.057 | 0.047 | 0.254 | 1.123 | 0.016 | 0.024 |
| Pond snails-Adults | 0.933 | 0.057 | 0.047 | 0.254 | 1.123 | 0.016 | 0.024 |
| Mussels-Children | 0.425 | 0.059 | 0.048 | 0.262 | 1.157 | 0.017 | 0.024 |
| Pond snails-Children | 0.961 | 0.135 | 0.421 | 0.098 | 1.216 | 0.025 | 0.042 |


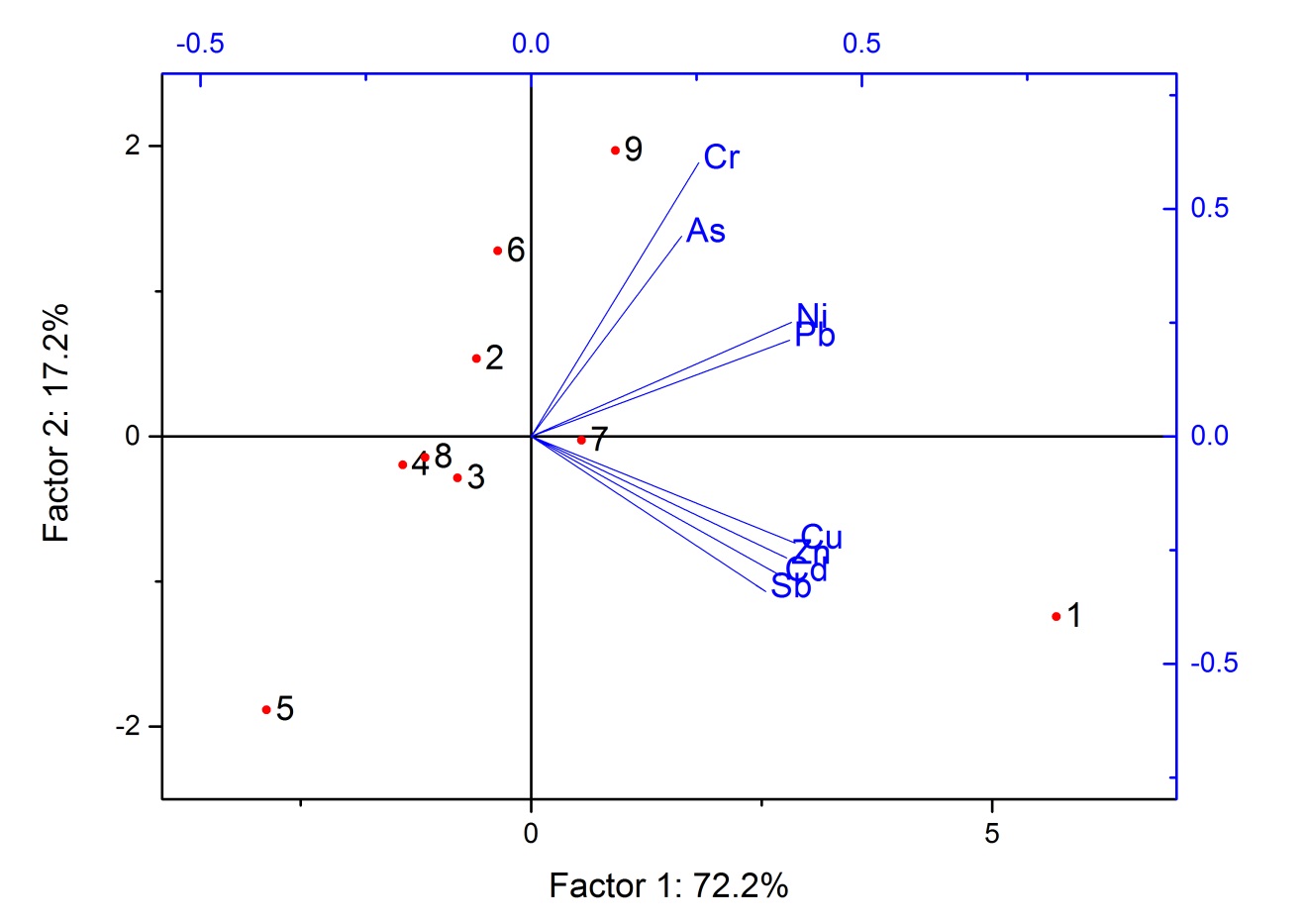


FIGURE S1. Biplot of trace metals and metalloids in sediments from eight sites (red dots) in Dianshan Lake.


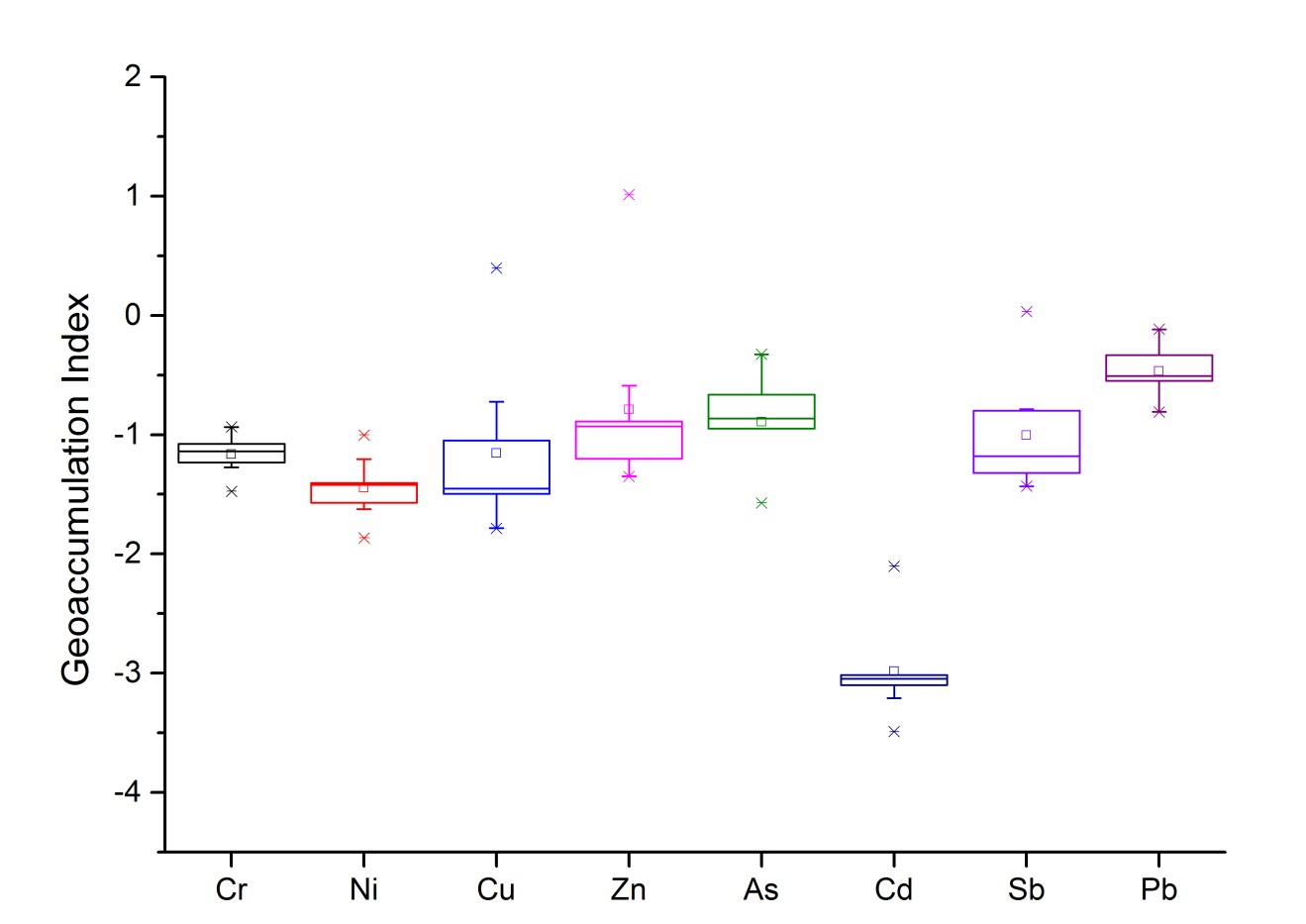


FIGURE S2. Geoaccumulation Index for trace elements in Dianshan Lake sediments.

**References**

Chien, L.-C., Hung, T.-C., Choang, K.-Y., Yeh, C.-Y., Meng, P.-J., Shieh, M.-J., et al. (2002). Daily intake of TBT, Cu, Zn, Cd and As for fishermen in Taiwan. *Science of the Total Environment, 285*(1-3), 177-185.

Hakanson, L. (1980). An ecological risk index for aquatic pollution control.a sedimentological approach. *Water Research, 14*(8), 975-1001.

Ji, Y., Feng, Y., Wu, J., Zhu, T., Bai, Z., & Duan, C. (2008). Using geoaccumulation index to study source profiles of soil dust in China. *Journal of Environmental Sciences, 20*(5), 571-578.

Long, E. R. (2006). Calculation and Uses of Mean Sediment Quality Guideline Quotients:  A Critical Review. *Environmental Science & Technology, 40*(6), 1726-1736.

MacDonald, D. D., Ingersoll, C. G., & Berger, T. A. (2000). Development and evaluation of consensus-based sediment quality guidelines for freshwater ecosystems. *Archives of Environmental Contamination and Toxicology, 39*(1), 20-31.

Muller, G. (1969). Index of geoaccumulation in sediments of the Rhine River. *Geological Journal, 2*(3), 108-118.

Tao, Y., Yuan, Z., Xiaona, H., & Wei, M. (2012). Distribution and bioaccumulation of heavy metals in aquatic organisms of different trophic levels and potential health risk assessment from Taihu lake, China. *Ecotoxicology and Environmental Safety, 81*, 55-64.

Turekian, K. K., & Wedepohl, K. H. (1961). Distribution of the Elements in Some Major Units of the Earth's Crust. *Geological Society of America Bulletin, 72*(2), 175.

USEPA (1989). Risk Assessment Guidance for Superfund Volume I Human Health Evaluation Manual (Part A). *United States Environmental Protection Agency*. Philadelphia, PA, WashingtonDC.

USEPA (1997). The Incidence And Severity Of Sediment Contamination In Surface Waters Of The United States. Volume 1: National Sediment Quality Survey. *United States Environmental Protection Agency*. Philadelphia, PA, WashingtonDC.

USEPA (2013). Regional Screening Level and Fish Ingestion Table. *United States Environmental Protection Agency*. Philadelphia, PA, WashingtonDC.

Zheng, N., Wang, Q., Zhang, X., Zheng, D., Zhang, Z., & Zhang, S. (2007). Population health risk due to dietary intake of heavy metals in the industrial area of Huludao City, China. *Science of the Total Environment, 387*(1-3), 96-104.
